# Web-Enhanced Tobacco Tactics With Telephone Support Versus 1-800-QUIT-NOW Telephone Line Intervention for Operating Engineers: Randomized Controlled Trial

**DOI:** 10.2196/jmir.3375

**Published:** 2014-11-20

**Authors:** Seung Hee Choi, Andrea H Waltje, David L Ronis, Devon Noonan, OiSaeng Hong, Caroline R Richardson, John D Meeker, Sonia A Duffy

**Affiliations:** ^1^Michigan State UniversityCollege of NursingEast Lansing, MIUnited States; ^2^University of MichiganSchool of NursingAnn Arbor, MIUnited States; ^3^Duke UniversitySchool of NursingDurham, NCUnited States; ^4^University of California: San FranciscoDepartment of Community Health SystemsSan Francisco, CAUnited States; ^5^University of Michigan Health SystemDepartment of Family MedicineAnn Arbor, MIUnited States; ^6^Ann Arbor VA Center for Clinical Management ResearchHealth Services Research and DevelopmentAnn Arbor, MIUnited States; ^7^University of MichiganSchool of Public HealthAnn Arbor, MIUnited States

**Keywords:** smoking cessation, intervention study, workplace, blue collar workers

## Abstract

**Background:**

Novel interventions tailored to blue collar workers are needed to reduce the disparities in smoking rates among occupational groups.

**Objective:**

The main objective of this study was to evaluate the efficacy and usage of the Web-enhanced “Tobacco Tactics” intervention targeting operating engineers (heavy equipment operators) compared to the “1-800-QUIT-NOW” telephone line.

**Methods:**

Operating engineers (N=145) attending one of 25 safety training sessions from 2010 through 2012 were randomized to either the Tobacco Tactics website with nurse counseling by phone and access to nicotine replacement therapy (NRT) or to the 1-800-QUIT-NOW telephone line, which provided an equal number of phone calls and NRT. The primary outcome was self-reported 7-day abstinence at 30-day and 6-month follow-up. The outcomes were compared using chi-square tests, t tests, generalized mixed models, and logistic regression models.

**Results:**

The average age was 42 years and most were male (115/145, 79.3%) and white (125/145, 86.2%). Using an intent-to-treat analysis, the Tobacco Tactics website group showed significantly higher quit rates (18/67, 27%) than the 1-800-QUIT NOW group (6/78, 8%) at 30-day follow-up (*P*=.003), but this difference was no longer significant at 6-month follow-up. There were significantly more positive changes in harm reduction measures (quit attempts, number of cigarettes smoked per day, and nicotine dependence) at both 30-day and 6-month follow-up in the Tobacco Tactics group compared to the 1-800-QUIT-NOW group. Compared to participants in the 1-800-QUIT NOW group, significantly more of those in the Tobacco Tactics website group participated in the interventions, received phone calls and NRT, and found the intervention helpful.

**Conclusions:**

The Web-enhanced Tobacco Tactics website with telephone support showed higher efficacy and reach than the 1-800-QUIT-NOW intervention. Longer counseling sessions may be needed to improve 6-month cessation rates.

**Trial Registration:**

Clinicaltrials.gov NCT01124110; http://clinicaltrials.gov/ct2/show/NCT01124110 (Archived by WebCite at http://www.webcitation.org/6TfKN5iNL).

## Introduction

Blue-collar workers (those who perform manual labor) are more likely to smoke than white-collar workers and are more likely to develop smoking-related diseases [1]. Despite the risks, blue-collar workers have limited access to smoking cessation interventions [2] and only half of construction workers were advised to quit smoking [3]. When provided with interventions, blue-collar workers are less likely to use proven tobacco cessation treatments compared to those of higher socioeconomic status (SES) [2]. Moreover, blue-collar workers do not benefit from worksite smoking bans and restrictions. While there is an understanding of factors that contribute to elevated tobacco use in blue-collar workers, little research has focused on cessation. Novel approaches to disseminate efficacious interventions are likely to reduce tobacco-related disparities and cancers among blue-collar workers [4].

One group of blue-collar workers, operating engineers (those who are responsible for the operation of heavy earth-moving equipment to construct buildings, bridges, and roads) showed a higher smoking rate [5]. Among workers in dusty occupations, such as operating engineers, smoking is particularly detrimental because of the exposure to occupational hazards, such as asbestos, cement dust, coal tar pitch, and diesel exhaust, which has a dose-response synergic effect with smoking to develop pulmonary diseases [6]. Thus, operating engineers are particularly at risk for smoking-related diseases, such as cardiovascular disease [7], pulmonary disease [7], as well as cancers of the lung [8], head and neck [9], and trachea and bronchus [10].

Our prior work with operating engineers has shown that 29% smoke [11] compared to 19% among the general population [12], over half are interested in quitting, and they have access to a computer during their regularly scheduled safety trainings [13]. Web-enhanced cessation interventions have been shown to reduce tobacco use [14-17], be more efficacious if they provide tailored messages [18], and enhance quit rates when in conjunction with NRTs [16,18,19]. To our knowledge, there are a few smoking cessation interventions targeting blue-collar workers [20,21], but none of them are Web-enhanced. The Tobacco Tactics website was built for operating engineers based on an efficacious face-to-face intervention [22]. The development of the website was described in detail in a previously published paper [23]. The specific aim of this paper was to compare the Tobacco Tactics website targeting operating engineers to the state-sponsored 1-800-QUIT-NOW telephone line on: (1) 30-day and 6-month self-reported quit rates, (2) 6-month cotinine levels, (3) number of quit attempts, (4) nicotine dependence, (5) number of cigarettes smoked/day, (6) smoking self-efficacy, (7) contacts with interventions, (8) medications used, (9) helpfulness of the interventions, and (10) willingness to recommend the interventions to others.

## Methods

### Design

The protocol of this study was described in a previously published manuscript [24]. In this randomized controlled trial (trial registration: ClinicalTrials.gov NCT01124110), operating engineers attending one of 25 safety training sessions from 2010 through 2012 were randomized either to the Tobacco Tactics website intervention or to the 1-800-QUIT-NOW state-supported telephone quit line. Since there was a high probability of cross-contamination within training sessions, randomization occurred at the training class level rather than individual level [17,25]. Institutional Review Board approval was received from the University of Michigan.

### Setting and Sample

At the Operating Engineers Local 324 Training Center, workers attending annual safety training sessions were invited to participate in this study. Inclusion criteria were operating engineers who were (1) older than 18 years of age, (2) current smokers, and (3) interested in participating in a cessation program. Exclusion criteria were operating engineers who were (1) non-English speaking (the interventions are only available in English), and (2) pregnant.

### Procedures

Operating engineers interested in the study were provided with an information sheet about the study and consent forms. Once participants completed a baseline survey, they were given time to make the first contact with the intervention. Training groups randomized into the Tobacco Tactics intervention group were provided with a computer with Internet access and those randomized into the 1-800-QUIT-NOW were offered a telephone at the training center.

Follow-up surveys were mailed at 30-days and 6-months asking about smoking status, covariates, and their opinions about the intervention. To increase response rates, those who did not return mail surveys were given the opportunity to complete the surveys on the phone. At 6-month follow-up, participants were also sent a NicAlert urinary cotinine test to return with their survey. Those who completed surveys received US $15 for the baseline survey, US $15 for the 30-day survey, and US $20 for the 6-month survey and cotinine test. Data were collected from 2010 through 2012 and analyzed in 2013.

### Description of the Tobacco Tactics Website Intervention

**Figure 1:**
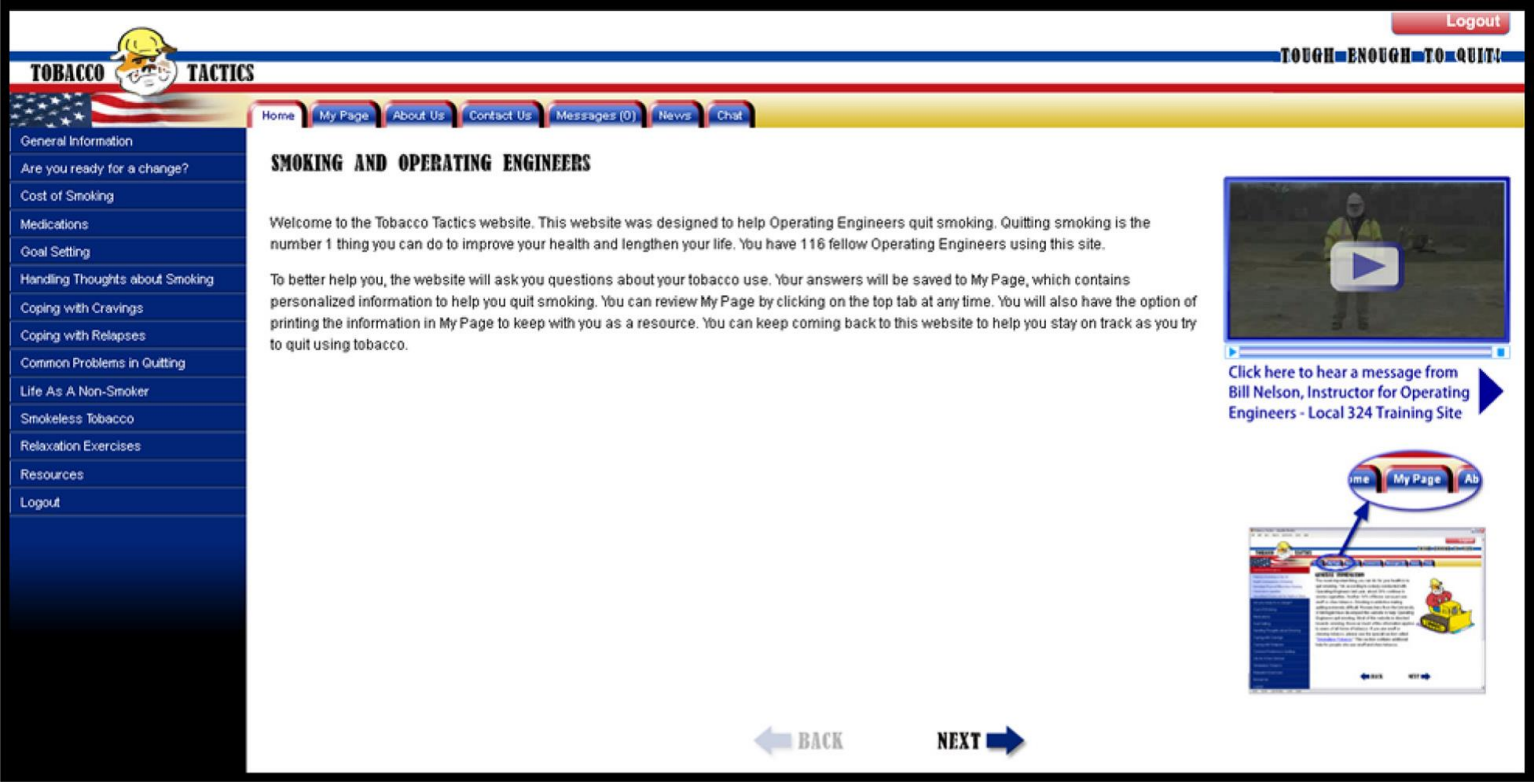
Screenshot of the intervention.

**Figure 2:**
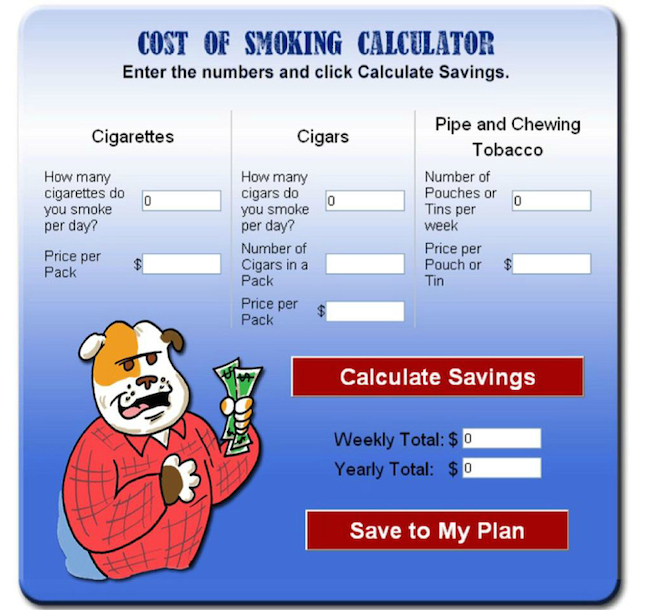
Screenshot of the intervention.

The development of the Tobacco Tactics website is described in detail in an earlier publication [23]. The Tobacco Tactics website contains humorous graphics tailored to operating engineers, tailored cessation feedback, and follow-up nurse counseling offered by multimedia options including phone and/or email, and/or e-community (Figure 1 and Figure 2). 

The content was written at an 8^th^ grade reading level and provided interactive cognitive behavioral therapy exercises including a self-assessment of tobacco habit, calculation of a nicotine dependence score, identification of smoker type, calculation of money savings, tips for prepare for quitting (eg, cleaning the car of cigarette butts, etc), a change plan work sheet, and strategies for coping with relapses. Additional interactive components provide mechanisms for tobacco users to assess their smoking habit, set a quit date, and monitor weekly progress.

Since peer support has been shown to enhance behavioral interventions [26], there was also a nurse-monitored e-community. A research nurse served as a group moderator for the e-community three times per week, answered questions, and posted questions to stimulate group discussion. Upon each log-off, participants were asked to answer a few brief questions about their tobacco habit, which resulted in a graph that showed their progress in quitting over time. Operating engineers were offered their choice of a full supply of over-the-counter nicotine patches, gum, lozenges, or a combination of these for highly addicted smokers.

Since studies have shown that telephone and nurse counseling is efficacious [27-29] and tailored telephone and regular postal mail cessation interventions have been found to be efficacious among construction workers [20], the nurse made follow-up telephone and/or email counseling contacts at 2, 7, 14, 21, and 30 days after the training. Follow-up contacts reinforced the initial website visit, promoted skill building, and monitored pharmacologic treatment.

### Description of the 1-800-QUIT-NOW Intervention

According to the recommendations for the design of control group conditions in clinical trials [30], the control group condition should be designed to be equivalent as much as possible on time spent, follow-up times, and attention given to participants. In keeping with the recommendations, the 1-800-QUIT-NOW intervention was chosen as a control condition since it was as equivalent as possible to the Tobacco Tactics Web-enhanced intervention in terms of baseline counseling from the study nurse, numbers of follow-up calls, and medications available. Participants randomized to the 1-800-QUIT-NOW were counseled by the study nurse to call and were given time to do so at their safety training class. The first time participants called the quit line, they received a personal coach who assisted them in setting a quit date and making an individualized quit plan, followed by up to five telephone coaching sessions around the caller’s quit date and free NRT (patches or gum), which were all equivalent to the Tobacco Tactics intervention.

### Measures

#### Dependent Variables

The primary dependent variable was self-reported 7-day point prevalence smoking cessation rates at 30-day and 6-month follow-up by asking the well-validated question, “Have you used any tobacco products in the past 7 days?” [31]. The secondary dependent variable was a cotinine-verified 6-month smoking status using a mailed urinary cotinine test kit. Urinary cotinine assessment has excellent reproducibility and high sensitivity (92%) and specificity (91%) for identifying non-smokers from smokers [32]. Using an intention-to-treat analysis, participants who were not available for follow-up or did not return the survey were considered smokers and those who did not return the cotinine test or who had an unreadable cotinine strip were considered to test positive for smoking for the biochemical confirmation analyses.

Harm reduction was assessed including (1) quit attempts for at least 24 hours, (2) (changes in) nicotine dependence, (3) (changes in) number of cigarettes smoked/day, and (4) (changes in) smoking self-efficacy. Nicotine dependence was assessed using the Fagerstrom Test for Nicotine Dependence (FTND) [33] and the self-efficacy was measured by the Smoking Self-Efficacy Questionnaire (SEQ-12) [34].

As a process evaluation, both interventions were evaluated in terms of (1) percent that had contacts with intervention, and (2) percent that used medications. Participants were asked to rate the interventions on a scale of 1 to 5 (higher scores were better) in terms of (1) helpfulness of the phone calls and NRTs (extremely unhelpful to extremely helpful), (2) opinion about the number of calls (far too many to far too few), (3) comfort asking questions, level of support provided, and willingness to recommend the interventions to others (strongly disagree to strongly agree), and (4) satisfaction with answers (extremely unsatisfied to extremely satisfied).

Those randomized to the Web-enhanced Tobacco Tactics intervention only were asked to rate components of the website on a scale of 1 to 5 (with higher scores were better). Ease of use, enjoyability, navigation, feedback from interactive exercises, and satisfaction were rated from strongly disagree to strongly agree. The interactive exercises of smoking assessment, reasons to quit, smoking log, smoking triggers, cigarette substitutes, and medication were rated from extremely unhelpful to extremely helpful. The home page, title, and pictures and illustrations were rated from very poor to excellent. This information was collected from an administrative component of the website, nurse logs of contacts, and survey data. Similar survey questions were asked of those randomized to the 1-800-QUIT-NOW quit line intervention.

#### Independent Variables

The main independent variable was the Tobacco Tactics Web-enhanced intervention versus the 1-800-QUIT-NOW intervention. Covariates that might influence smoking were also examined. Alcohol use was measured by the Alcohol Use Disorders Identification Test (AUDIT) with scores of 8 or higher indicating problem drinking [35]. Social support was measured by the ENRICHED Social Support Instrument [36] and the Perceived Stress Scale was used to assess stress [37]. Depressive symptoms were assessed by the Center for Epidemiologic Studies Depression Scale (CES-D) with scores of 16 or higher indicating significant depressive symptoms [38]. Medical comorbidities were assessed by the validated measure [39] and questions about demographics were asked.

### Data Analysis

Descriptive statistics were computed for all variables. The equivalence of the two groups at baseline was tested using χ^2^ tests or Fisher’s exact tests for categorical variables and two-tailed *t* tests for quantitative variables. To compare the two interventions on efficacy and usage, χ^2^ tests or Fisher’s exact tests and *t* tests were conducted using an intention-to-treat analysis in which non-responders were considered smokers. These analyses for quit rates were repeated controlling for differences between the groups using logistic regression. Since the randomization occurred at the training group level, to test for cluster effects, tests of heterogeneity for smoking status at 30-day and 6-month follow-up were performed using mixed models. Since the sample size was small, if there was no significant heterogeneity, final analyses were conducted with chi-square tests or *t* tests not adjusting the clustering by training group. In all analyses, an alpha level of .05 two-tailed was used as the criterion for significance. Sample size may vary for selected variables due to missing data.

## Results

### Recruitment and Retention

**Figure 3:**
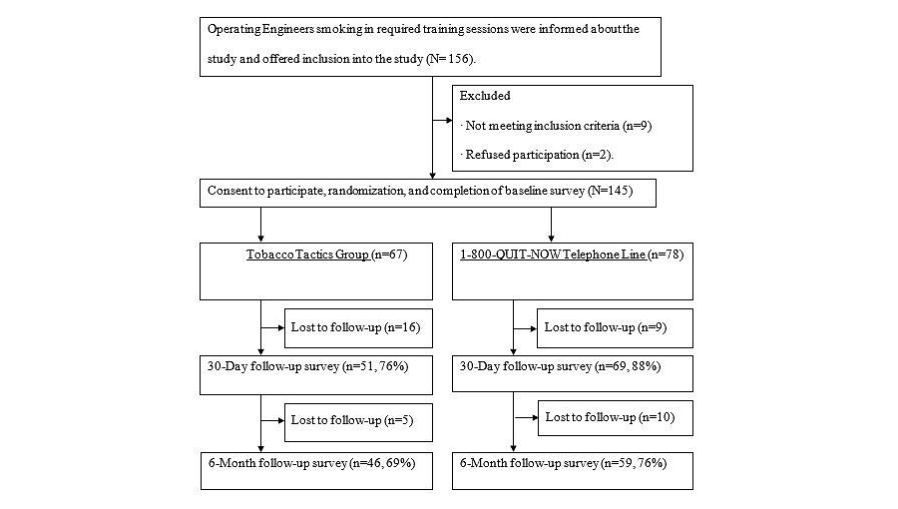
CONSORT Flowdiagram.

Over the course of 3 years (2010 to 2012), 25 training groups were randomized with an average size of 6 participants per group, leading to 67 participants in the website group and 78 participants in the 1-800-QUIT-NOW group (N=145). A total of 83% of the sample (120/145, 82.8%) completed the 30-day survey and 73% (105/145, 72.4%) completed the 6-month survey. Those who were not thinking of quitting within the next 30 days (*P*=.029) and reported higher numbers of snuff used in the past month (*P*=.003) were more likely to drop out before 30-day follow-up. Those who reported that they were not thinking of quitting (*P*=.021), were veterans (*P*=.044), and were without hypertension (*P*=.033) were more likely to drop out before 6-month follow-up. A CONSORT diagram can be found in Figure 3.

### Description of the Sample and Equivalency of the Groups

The description of the sample can be seen in Table 1. The average age of the operating engineers was 42 (SD 9.5) years, most were male (115/145, 79.3%) and white (125/145, 86.2%). Just over half were married (80/145, 55.9%) and had a high school education or less (88/145, 61.1%). The most common comorbidities were high blood pressure (29/145, 20.4%), psychiatric problems (21/145, 14.7%), substance abuse problems (16/145, 11.2%), and lung disease (14/145, 10.1%). A total of 45.1% screened positive for problem drinking (60/133) and 35.4% (51/145) screened positive for significant depressive symptoms. The mean perceived stress score was 24.3 (SD 6.8) (range 9-43), which is comparable to mean scores in other populations such as students and community samples [37]. Just under one-third (42/145, 29.0%) of operating engineers reported low social support. While the groups were equivalent on most factors at baseline, subjects in the website group showed significantly higher body mass index (BMI) (*P*=.029) and less motivation to quit smoking (*P*=.042) compared to those in the 1-800-QUIT NOW group.

**Table 1:** Baseline characteristics of the Tobacco Tactics website and 1-800-QUIT-NOW participants.

Group		All (N=145)	Website (n=67)	1-800-QUIT-NOW (n=78)	*P* value
		mean (SD; range) or n (%)			
Age, years		42.0 (9.5; 20-61)	42.1 (9.3; 23-60)	41.8 (9.7; 20-61)	.837
BMI^a^		29.0 (5.7; 20–53)	30.1 (6.0; 21-53)	28.1 (5.3; 20-44)	.029
Years of regular smoking (n_I_=65; n_C_=77)^b^		21.5 (10.1; 1-47)	22.0 (10.3; 1-45)	21.2 (10.0; 1-47)	.651
Number of cigarettes smoked per day (n_I_=64; n_C_=74)		20.9 (9.9; 1-50)	22.2 (8.4; 3-50)	19.8 (11.0; 1-50)	.162
Nicotine dependence (FTND^c^) (n_I_=63; n_C_=77)		4.7 (2.6; 0-10)	5.1 (2.4; 0-9)	4.4 (2.7; 0-10)	.149
Smoking self-efficacy (SEQ-12^d^)		32.7 (10.5; 12-60)	33.5 (10.8; 12-60)	32.0 (10.3; 12-56)	.401
Perceived stress scale score		24.3 (6.8; 9-43)	24.3 (6.6; 9-43)	24.4 (6.9; 10-42)	.972
**Sex**					.063
	Male	115 (79.3)	58 (86.6)	57 (73.1)	
	Female	30 (20.7)	9 (13.4)	21 (26.9)	
Hispanic or Latino		6 (4.1)	2 (3.0)	4 (5.1)	.686
**Race**					.339
	White	125 (86.2)	60 (89.6)	65 (83.3)	
	Non-white (all others)	20 (13.8)	7 (10.4)	13 (16.7)	
**Marital status (n**_**I**_ **=66; n**_**C**_ **=77)**					.738
	Married	80 (55.9)	38 (57.6)	42 (54.5)	
	Not married	63 (44.1)	28 (42.4)	35 (45.5)	
**Educational level (n**_**I**_ **=66; n**_**C**_ **=78)**					.609
	High school or less than high school	88 (61.1)	42 (63.6)	46 (59.0)	
	More than high school	56 (38.9)	24 (36.4)	32 (41.0)	
Do you live alone? (n_I_=64; n_C_=78)		21 (14.8)	8 (12.5)	13 (16.7)	.636
**Medical comorbidities**					
	High blood pressure (n_I_=65; n_C_=77)	29 (20.4)	13 (20.0)	16 (20.8)	1.000
	Psychiatric problems (n_I_=66; n_C_=77)	21 (14.7)	9 (13.6)	12 (15.6)	.815
	Substance abuse problems (n_I_=66; n_C_=77)	16 (11.2)	7 (10.6)	9 (11.7)	1.000
	Lung disease (n_I_=63; n_C_=75)	14 (10.1)	7 (11.1)	7 (9.3)	.782
	Diabetes (n_I_=65; n_C_=76)	7 (5.0)	5 (7.7)	2 (2.6)	.248
	Heart disease (n_I_=64; n_C_=76)	7 (5.0)	4 (6.2)	3 (3.9)	.702
	Arthritis (n_I_=62; n_C_=74)	7 (5.1)	2 (3.2)	5 (6.8)	.454
	Cancer (n_I_=66; n_C_=77)	6 (4.2)	2 (3.0)	4 (5.2)	.678
	Stroke (n_I_=64; n_C_=77)	1 (0.7)	0 (0.0)	1 (1.3)	1.000
Alcohol problem (AUDIT ≥8) (n_I_=63; n_C_=70)		60 (45.1)	26 (41.3)	34 (48.6)	.486
Depressed (CES-D≥16) (n_I_=66; n_C_=78)		51 (35.4)	18 (27.3)	33 (42.3)	.080
Low social support		42 (29.0)	22 (32.8)	20 (25.6)	.364
**Thinking of quitting**					
	Yes, within next 30 days	76 (52.4)	32 (47.8)	44 (56.4)	.042
	Yes, within next 6 months	64 (44.1)	30 (44.8)	34 (43.6)	
	No	5 (3.4)	5 (7.5)	0 (0.0)	
**Used in past month (yes/no)**					
	Cigars	17 (11.7)	7 (10.4)	10 (12.8)	.854
	Pipes	2 (1.4)	1 (1.5)	1 (1.3)	1.000
	Cigarillos	2 (1.4)	2 (3.0)	0 (0.0)	.411
	Snuff	21 (14.5)	7 (10.4)	14 (17.9)	.297
Smoking of closest person (n_I_=56; n_C_=64)		57 (47.5)	24 (42.9)	33 (51.6)	.365
Ever tried to quit		126 (86.9)	56 (83.6)	70 (89.7)	.328

^a^BMI: body mass index

^b^n_I_: intervention sample; n_C_: control sample

^c^FTND: Fagerstrom Test for Nicotine Dependence

^d^SEQ-12: Smoking Self-Efficacy Questionnaire

### Efficacy of the Interventions

The differences in 7-day point prevalence quit rates between the groups can be seen in Table 2. The Tobacco Tactics website group had significantly higher quit rates (18/67, 27%) than the 1-800-QUIT-NOW group (6/78, 8%) at 30-day follow-up (*P*=.003). However, the differences were not significant at 6-month follow-up (12%, 8/67, vs 12%, 9/78). Repeating these analyses controlling for BMI and motivation to quit smoking, which differed across the two groups at baseline, produced similar results; the odds of non-smoking in the website group was 4.8 times as great as in the 1-800-QUIT NOW group (OR 4.8, *P*=.003). Tests for heterogeneity for smoking status at 30-day and 6-month follow-ups among the training groups were not significant and hence training group was not controlled for in the analyses. Only 20.7% (30/145) of the operating engineers returned cotinine strips, hence cotinine-verified quit rates could not be determined.

Compared to the 1-800-QUIT-NOW group, more operating engineers in the Tobacco Tactics website group were able to quit for at least 24 hours: 69% (36/67) vs 23% (16/78) at 30-day follow-up (*P*<.001) and 70% (32/67) vs 43% (26/78) at 6-month follow-up (*P*=.010), yet the numbers of quit attempts were not significantly different between the groups. Moreover, the website group showed greater reductions in nicotine dependence (*P*=.006 at 30-day follow-up and *P*=.014 at 6-month follow-up) and the number of cigarettes smoked per day (*P*<.001 at 30-day follow-up and *P*=.017 at 6-month follow-up). Participants in the website group smoked significantly fewer cigarettes per day: 12.4 vs 17.7 at 30-day follow-up (*P*=.020) and 13.6 vs 19.1 at 6-month follow-up (*P*=.046). Similarly, those in the website group reported higher levels of smoking self-efficacy (*P*=.003) and greater increases in smoking self-efficacy (*P*=.010) at 30-day follow-up than those who were in the 1-800-QUIT-NOW group.

**Table 2:** Tobacco use among the Tobacco Tactics website and 1-800-QUIT-NOW participants.

Surveys completed	Baseline			30-day follow-up			6-month follow-up		
Group	Website (n=67)	1-800-QUIT-NOW (n=78)	*P* value	Website (n=67)	1-800-QUIT-NOW (n=78)	*P* value	Website (n=67)	1-800-QUIT-NOW (n=78)	*P* value
Non-smoking, n (%)				18 (27)	6 (8)	(n=145) *P*=.003	8 (12)	9 (12)	(n=145) *P*=1.000
Able to quit 24 hours, n (%)				36 (69)	16 (23)	(n=121) *P*<.001	32 (70)	26 (43)	(n=106) *P*=.010
Nicotine dependence score, mean (SD)	5.1 (2.4)	4.4 (2.7)	(n=140) *P*=.149	2.9 (2.7)	3.5 (2.8)	(n=103) *P*=.262	3.8 (2.8)	4.2 (2.9)	(n=74) *P*=.614
Changes in nicotine dependence, mean (SD)				−2.3 (3.0)	−0.8 (2.1)	(n=98) *P*=.006	−1.5 (2.3)	−0.2 (2.0)	(n=71) *P*=.014
Number of cigarettes/day, mean (SD)	20.4 (12.9)	18.3 (12.8)	(n=145) *P*=.336	12.4 (10.3)	17.7 (13.4)	(n=121) *P*=.020	13.6 (11.7)	19.1 (16.4)	(n=106) *P*=.046
Changes in number of cigarettes/day, mean (SD)				−9.2 (14.7)	0.3 (14.1)	(n=121) *P*<.001	−6.6 (17.6)	1.0 (14.5)	(n=106) *P*=.017
Number of quit attempts, mean (SD)				5.3 (7.8)	6.1 (7.8)	(n=43) *P*=.776	6.4 (7.8)	5.0 (7.1)	(n=44) *P*=.537
Smoking self-efficacy, mean (SD)	33.5 (10.8)	32.0 (10.3)	(n=145) *P*=.401	39.1 (10.0)	31.5 (10.4)	(n=76) *P*=.003	33.8 (12.6)	31.9 (12.1)	(n=70) *P*=.513
Changes in smoking self-efficacy, mean (SD)				9.5 (12.2)	1.1 (13.1)	(n=75) *P*=.010	1.5 (13.3)	0.3 (15.0)	(n=68) *P*=.748

### Usage of the Interventions

Multimedia Appendix 1 compares the usage of the two interventions. Significantly more participants in the website group participated in the intervention than those in the 1-800-QUIT-NOW group (*P*<.001). The majority of the participants (66/67, 99%) in the website group visited the Tobacco Tactics website at least once and the average was 2.7 (SD 3.7) visits. Compared to the 1-800-QUIT-NOW group (11/78, 14%), significantly more participants in the website group (60/67, 90%) participated in phone counseling (*P*<.001). While 70% (48/67) of the website group received any kind of NRTs, only 5% (4/78) of the 1-800-QUIT-NOW group received NRT (*P*<.001); patches, 40% (29/67) vs 3% (2/78) (*P*<.001), gum, 60% (40/67) vs 1% (1/78) (*P*<.001), lozenges, 9% (6/67) vs 0% (0/78) (*P*=.009), and patches and gum or lozenges, 36% (24/67) vs 0% (0/78) (*P*<.001).

Participants were asked to rate components of both interventions on a scale of 1 to 5 (higher scores were better). Overall helpfulness of the phone calls was rated significantly higher in the website group than the 1-800-QUIT-NOW group (*P*=.023). There was no significant difference in the participants’ opinions about the number of calls received (3.3 compared to 3.1, *P*=.604). However, participants in the website group reported more comfort with asking questions (*P*=.010), more satisfaction with the answers provided by the counselors (*P*=.003), and felt more supported (*P*<.001) than those in the 1-800-QUIT-NOW group. There was no difference between the groups in terms of tendency to recommend the intervention to someone else (*P*=.171).

Individuals that were randomized to the Web-enhanced Tobacco Tactics intervention were asked to rate specific components of the website on a scale of 1 to 5, with higher scores being better (Table 3). The majority (33/44, 75%) thought that it was overall recommendable. The lowest rated items were “helpful feedback” (20/44, 47%), “medication assessment” (21/44, 48%), and “smoking log” (16/44, 36%). Additional analysis (not shown in Tables) revealed the number of contacts with the website was not correlated with quit rates. However, the higher number of phone calls the Tobacco Tactics intervention participants received by the study nurse was correlated with higher cessation rates (*P*<.001). About 40% (27/67) attended the e-community chat room. The most common subjects discussed in the chat room included (1) checking/evaluating quitting process, (2) suggesting /sharing /encouraging strategies for smoking cessation, (3) NRTs, and (4) withdrawal symptoms.

**Table 3:** Percent of respondents that rated the Tobacco Tactics website as 4 or 5 on a 5-point scale, with higher numbers being better (n=44).

Rating		n (%)
**General evaluation of Tobacco Tactics: responded as Strongly Agree or Agree**		
	Overall recommendable	33 (75)
	Easy to navigate	30 (68)
	Easy to read and understand	26 (67)
	Easy to use of interactive exercises	27 (61)
	Overall satisfactory	26 (59)
	Enjoyable to visit	22 (50)
	Helpful feedback	20 (47)
**Exercises of Tobacco Tactics: responded as Extremely Helpful or Somewhat Helpful**		
	Reasons to quit	26 (59)
	Smoking self-assessment	25 (57)
	Cigarette substitutes	24 (55)
	Smoking triggers	22 (50)
	Medication assessment	21 (48)
	Smoking log	16 (36)
**Design of Tobacco Tactics: responded as Excellent or Good**		
	Main page	26 (59)
	Title	24 (55)
	Pictures and illustrations	22 (50)

## Discussion

### Principal Findings

The Tobacco Tactics Web-enhanced intervention for operating engineers produced three times higher quit rates at 30-day follow-up than the 1-800-QUIT-NOW quit line. Compared to other studies, the quit rate of 27% (18/67) is at the higher end of Internet-based smoking cessation interventions that reported successful results, which range from 11.0% to 27.7% [18,40,41]. There are several factors that led to the success of the Tobacco Tactics intervention. First, the Tobacco Tactics was developed to target operating engineers, featuring humorous cartoon characters of this population and containing tailored cessation feedback, which have been shown to increase quit rates [19,42]. All the content was written at an 8^th^ grade reading level and was easy to understand, which was critical since almost two-thirds had a high school education or less.

Second, the Tobacco Tactics website was available anytime and accessed as frequently as desired. All but one of the operating engineers that were randomized to the intervention group was able to explore the website at least once at the training site and many operating engineers repeated their visits up to 26 times.

Third, recruiting participants during their regularly scheduled safety trainings, which they attend each winter, may have enhanced quit rates as they were given on-the-spot intervention. Even though individuals randomized to the 1-800-QUIT-NOW were given the same amount of time to make a first contact, the majority of them did not make phone calls and the low-reach of the quit line intervention is consistent with previous studies [43]. As a result, the Tobacco Tactics website group received six times more phone counseling and 14 times more NRTs than the 1-800-QUIT-NOW group, which led to higher quit rates.

Unfortunately, the higher quit rates in the Tobacco Tactics Web-enhanced intervention group were not sustained at 6-month follow-up, which is consistent with a previous study with similar population [21]. A longer follow-up period may be needed to increase sustainability [44]. Previous studies have shown that obesity and concerns about weight gain can cause quit attempts to fail [45,46] and this may partially explain the non-significant quit rate at 6-month follow-up since the intervention group was significantly heavier than the control group, although an analysis controlling for obesity did not show different results. Nonetheless, future interventions may need to combine behavior change strategies targeting weight loss with those targeting smoking cessation [47].

Yet even among continuing smokers, compared to the 1-800-QUIT-NOW control group, those in the website group showed a significant reduction in number of cigarettes smoked per day and a reduction in nicotine dependence, suggesting that the Tobacco Tactics Web-enhanced intervention had a significant effect on harm reduction. Several studies acknowledged that the number of cigarettes smoked per day had a dose-response relationship with heart and lung disease [48,49] and that harm reduction decreases the risk of smoking-related diseases possibly through reductions in tobacco toxin exposure, such as carbon monoxide [50]. Since the average smoker makes seven quit attempts before actually quitting [51] and past quit attempts strongly predicted future quit attempts [52], those operating engineers who did not quit made substantial progress in the direction of quitting.

Over three-quarters of respondents randomized to the Web-enhanced Tobacco Tactics intervention strongly agreed or agreed that it was recommendable to others. Yet there were components of the intervention that were rated lower. The Web-based medication assessment was among the lower-rated items, suggesting that a conversation may be needed to figure out the best medications for an individual based on their smoking habit. While there were interactive exercises that gave feedback, feedback was among the lower-rated items suggesting that a website cannot suffice for personal contact. Moreover, there was a positive correlation between number of phone calls in the intervention group and quit rates. Social support has been shown to enhance smoking cessation interventions [26,53]. While the e-community chat room provided some social support, just under half participated in the chat room and the number of calls received was correlated with quitting, albeit those participating in the calls may be more motivated to quit.

### Limitations

The sample was primarily white and male, but was representative of the sample of operating engineers in Michigan [11,54]. The sample size was a bit small to control for clustering of training groups, although this was less of a problem since tests for heterogeneity for smoking status at 30-day and 6-month follow-up among the training groups were not significant. Only one-third of the operating engineers completed the biochemical validation and we anecdotally heard that they felt biochemical verification was offensive, which is a limitation of the study, although our prior work has shown high sensitivity and specificity between self-report and biochemical verification in other populations of primarily male smokers [55]. Even though training groups were randomized, there were baseline differences between the groups (such as BMI and motivation to quit), yet controlling for these factors in the analysis did not change the results. The Tobacco Tactics Web-enhanced intervention was composed of three parts (Tobacco Tactics website, phone counseling, and NRTs) and was tested as a whole, therefore the specific components of the Tobacco Tactics that led to success in smoking cessation and harm reduction could not be determined, which is often the case with multi-component interventions. The multi-component Tobacco Tactics intervention was provided by one study nurse, perhaps causing an intervener effect by increasing engagement in the intervention and impact on the outcomes, which may influence construct and the internal validity [56].

### Conclusions

The Web-enhanced Tobacco Tactics intervention for operating engineers showed a significantly higher efficacy and higher reach at 30-day follow-up compared to the 1-800-QUIT-NOW quit line. Among those who did not quit at 6-month follow-up, there were significant increases in harm reduction in the intervention group compared to the 1-800-QUIT-NOW telephone line. Web-enhanced smoking cessation interventions are cost effective [19] in that once a website is built, the cost of reaching 1 million tobacco users is not much more than reaching 1000 tobacco users [57]. Without considering the cost of medications, Web-enhanced smoking cessation interventions have been shown to cost less than US $1 per smoker, which is a lot less than either telephone counseling or print product interventions [19]. Therefore, the Web-enhanced Tobacco Tactics smoking cessation intervention has the potential to have high reach and efficacy at a low cost. Based on our results, we will revise our strategy and explore the possibility of dissemination via the operating engineers National Training Center, which services North America (including the United States, Mexico, and Canada).

## Multimedia Appendix 1

Usage and satisfaction with Tobacco Tactics and 1-800-QUIT-NOW interventions.

## Multimedia Appendix 2

CONSORT-EHEALTH checklist V1.6.2 [58].

## Abbreviations

AUDIT: Alcohol Use Disorders Identification Test
BMI: body mass index
CES-D: Center for Epidemiologic Studies Depression Scale
FTND: Fagerstrom Test for Nicotine Dependence
NRT: nicotine replacement therapy
RCT: randomized controlled trial
SES: socioeconomic status
SEQ-12: Smoking Self-Efficacy Questionnaire
