# Supplementary material for: Web-Enhanced Tobacco Tactics With Telephone Support Versus 1-800-QUIT-NOW Telephone Line Intervention for Operating Engineers: Randomized Controlled Trial
Source: J Med Internet Res. 2014 Nov 20;16(11):e255. doi: 10.2196/jmir.3375 (PMC4260077; doi:10.2196/jmir.3375)
Supplement: Supplementary file 1 [file jmir_v16i11e255_app1.pdf]

Usage and Satisfaction with Tobacco Tactics and 1-800-QUIT-NOW Interventions

Group	Website Frequency (%) (n=67)	1-800-QUIT-NOW Frequency (%) (n=78)	P-Value
Contacts with the intervention (n=126)	67 (100)	11 (14)	**<.001**
Visits to the website	66 (99)	N/A	
Received phone calls (n=145)	60 (90)	11 (14)	**<.001**
NRTs (n=145)	48 (70)	4 (5)	**<.001**
Patches	29 (40)	2 (3)	**<.001**
Gum	40 (60)	1 (1)	**<.001**
Lozenges	6 (9)	0 (0)	**<.009**
Patches and gum or lozenges	24 (36)	0 (0)	**<.001**
	Mean (SD) (n=51)	Mean(SD) (n=11)	P-Value
Visits to the website (n=67)	2.7 (3.7) Range: 0-26	NA	
Helpfulness of Phone Calls (n=62)	4.0 (1.0)	3.3 (0.7)	**.023**
Opinion about number of calls Received (n=47)	3.3 (1.1)	3.1 (1.0)	.604
Comfort asking questions (n=62)	4.2 (0.8)	3.5 (0.7)	**.010**
Satisfaction with the answers (n=62)	4.3 (0.7)	3.6 (0.5)	**.003**
Level of support provided (n=62)	4.3 (0.7)	3.5 (0.5)	**<.001**
Helpfulness of NRTs (n=41)	4.0 (1.0)	3.8 (0.5)	.693
Willingness to recommend to someone else (n=62)	4.0 (0.6)	3.7 (1.0)	.171
