# Supplementary material for: Web-Enhanced Tobacco Tactics With Telephone Support Versus 1-800-QUIT-NOW Telephone Line Intervention for Operating Engineers: Randomized Controlled Trial
Source: J Med Internet Res. 2014 Nov 20;16(11):e255. doi: 10.2196/jmir.3375 (PMC4260077; doi:10.2196/jmir.3375)
Supplement: Supplementary file 2 [file jmir_v16i11e255_app2.pdf]

CONSORT-EHEALTH Checklist V1.6.2 Report

(based on CONSORT-EHEALTH V1.6), available at [http://tinyurl.com/consort-ehealth-v1-6].

3375

Date completed
3/4/2014 12:31:50
by
Seung Hee Choi

Randomized Controlled Trial of the Web-based Tobacco Tactics with Telephone Support versus 1-800-QUIT-NOW Telephone Line among Operating Engineers

TITLE

1a-i) Identify the mode of delivery in the title
"Randomized Controlled Trial of the Web-based Tobacco Tactics with Telephone Support versus 1-800-QUIT-NOW Telephone Line among Operating Engineers "

1a-ii) Non-web-based components or important co-interventions in title
"Randomized Controlled Trial of the Web-based Tobacco Tactics with Telephone Support versus 1-800-QUIT-NOW Telephone Line among Operating Engineers "

1a-iii) Primary condition or target group in the title
"Randomized Controlled Trial of the Web-based Tobacco Tactics with Telephone Support versus 1-800-QUIT-NOW Telephone Line among Operating Engineers "

ABSTRACT

1b-i) Key features/functionalities/components of the intervention and comparator in the METHODS section of the ABSTRACT
"nurse phone counseling"

1b-ii) Level of human involvement in the METHODS section of the ABSTRACT
One hundred forty five smokers were randomized to either the Tobacco Tactics website with nurse phone counseling and access to nicotine replacement therapy (NRT) or to the 1-800-QUIT-NOW telephone line which provided an equal number of phone calls and NRT. "

1b-iii) Open vs. closed, web-based (self-assessment) vs. face-to-face assessments in the METHODS section of the ABSTRACT
"on Operating Engineers attending one of 25 safety training sessions from 2010 through 2012"

1b-iv) RESULTS section in abstract must contain use data
mentioned in "Methods section" not in "Result section": "One hundred forty five smokers were randomized "

1b-v) CONCLUSIONS/DISCUSSION in abstract for negative trials
NA

INTRODUCTION

2a-i) Problem and the type of system/solution
"there are a few smoking cessation interventions targeting blue-collar workers"

2a-ii) Scientific background, rationale: What is known about the (type of) system
"Web-based cessation interventions have been shown to reduce tobacco use, be more efficacious if they provide tailored messages, and enhance quit rates when in conjunction with NRTs."

METHODS

3a) CONSORT: Description of trial design (such as parallel, factorial) including allocation ratio
"The specific aim of this paper was to compare the Tobacco Tactics website targeting Operating Engineers to the state sponsored 1-800-QUIT-NOW telephone line on: a) 30-day and 6-month self-reported quit rates; b) 6-month cotinine levels; c) number of quit attempts; d) nicotine dependence; e) number of cigarettes smoked/day; f) smoking self-efficacy; g) contacts with interventions; h) medications used; i) helpfulness of the interventions; and j) willingness to recommend the interventions to others."

3b) CONSORT: Important changes to methods after trial commencement (such as eligibility criteria), with reasons
NA (we did not change any trial commencement).

3b-i) Bug fixes, Downtimes, Content Changes
NA (we did not change any trial commencement).

4a) CONSORT: Eligibility criteria for participants
"Inclusion criteria were Operating Engineers who (1) were older than 18 years of age, (2) were current smokers, and (3) were interested in participating in a cessation program. Exclusion criteria were Operating Engineers who (1) were non-English speaking (the interventions are only available in English), and (2) were pregnant."

4a-i) Computer / Internet literacy
Not mentioned.

4a-ii) Open vs. closed, web-based vs. face-to-face assessments:
"At the Operating Engineers Local 324 Training Center, workers attending annual safety training sessions were invited to participate in this study"

4a-iii) Information giving during recruitment
"Operating Engineers interested in the study were provided with an information sheet about the study and consent forms"

4b) CONSORT: Settings and locations where the data were collected
"Training groups randomized into the Tobacco Tactics intervention group were provided with a computer with internet access and those randomized into the 1-800-QUIT-NOW were offered a telephone at the training center"

4b-i) Report if outcomes were (self-)assessed through online questionnaires
"Follow-up surveys were mailed at 30-days and 6-months"
"To increase response rates, those who did not return mail surveys were given the opportunity to complete the surveys on the phone"
4b-ii) Report how institutional affiliations are displayed
Not mentioned
5) CONSORT: Describe the interventions for each group with sufficient details to allow replication, including how and when they were actually administered
5-i) Mention names, credential, affiliations of the developers, sponsors, and owners
Not mentioned directly. But we stated that "The development of the website was described in detail a previously published paper"

Ewing, L., et al., Development of the Tobacco Tactics logo: From thumb prints to press. Tobacco Induced Diseases, 2012. 10(Suppl 6)
5-ii) Describe the history/development process
directed readers to the previous paper. "The development of the website was described in detail a previously published paper"

Ewing, L., et al., Development of the Tobacco Tactics logo: From thumb prints to press. Tobacco Induced Diseases, 2012. 10(Suppl 6)
5-iii) Revisions and updating
We described how to build the website in the previous manuscript.
Ewing, L., et al., Development of the Tobacco Tactics logo: From thumb prints to press. Tobacco Induced Diseases, 2012. 10(Suppl 6)
5-iv) Quality assurance methods
We described how to build the website in the previous manuscript.
Ewing, L., et al., Development of the Tobacco Tactics logo: From thumb prints to press. Tobacco Induced Diseases, 2012. 10(Suppl 6)
5-v) Ensure replicability by publishing the source code, and/or providing screenshots/screen-capture video, and/or providing flowcharts of the algorithms used
We provided this information in the previous paper.
Ewing, L., et al., Development of the Tobacco Tactics logo: From thumb prints to press. Tobacco Induced Diseases, 2012. 10(Suppl 6)
5-vi) Digital preservation
We provided the URL in the previous paper.
Ewing, L., et al., Development of the Tobacco Tactics logo: From thumb prints to press. Tobacco Induced Diseases, 2012. 10(Suppl 6)
5-vii) Access
"Training groups randomized into the Tobacco Tactics intervention group were provided with a computer with internet access and those randomized into the 1-800-QUIT-NOW were offered a telephone at the training center."
5-viii) Mode of delivery, features/functionalities/components of the intervention and comparator, and the theoretical framework
We described the components of the interventions in method section, (description of the Tobacco Tactics Website Intervention, Description of the 1-800-QUIT-NOW intervention)
5-ix) Describe use parameters
We evaluated the use of the interventions (contacts with the intervention, visits to the website, received phone calls, use of NRTs.)
5-x) Clarify the level of human involvement
"there was also a nurse-monitored e-community. A research nurse served as a group moderator for the e-community"
5-xi) Report any prompts/reminders used
"the Tobacco Tactics website intervention was enhanced with follow-up nurse telephone and/or email counseling contacts at 2, 7, 14, 21, and 30 days after the training. Follow-up contacts reinforced the initial website visit, promoted skill building, and monitored pharmacologic treatment."
5-xii) Describe any co-interventions (incl. training/support)
"Since peer support has been shown to enhance behavioral interventions, there was also a nurse-monitored e-community."
"Since studies have shown that telephone and nurse counseling is efficacious, and tailored telephone and regular postal mail cessation interventions have been found to be efficacious among construction workers, the Tobacco Tactics website intervention was enhanced with follow-up nurse telephone"
6a) CONSORT: Completely defined pre-specified primary and secondary outcome measures, including how and when they were assessed
The primary dependent variable was self-reported 7-day point prevalence smoking cessation rates at 30-day and 6-month follow-up." "The secondary dependent variable was a cotinine-verified 6-month smoking status using a mailed urinary cotinine test kit" "Harm reduction was assessed including (1) quit attempts for at least 24 hours, (2) (changes in) nicotine dependence, (3) (changes in) number of cigarettes smoked/day, and (4) (changes in) smoking self-efficacy."
"This information was collected from an administrative component of the website, nurse logs of contacts, and survey data. "
6a-i) Online questionnaires: describe if they were validated for online use and apply CHERRIES items to describe how the questionnaires were designed/deployed
Did not mentioned.
6a-ii) Describe whether and how “use” (including intensity of use/dosage) was defined/measured/monitored

"This information was collected from an administrative component of the website,
nurse logs of contacts, and survey data."

6a-iii) Describe whether, how, and when qualitative feedback from participants was obtained

"The secondary outcome was...... evaluations of the interventions"
"The interventions were also evaluated in terms of: a) contacts with intervention; b) medications used; c) helpfulness of the interventions on a 5-point scale ranging from extremely unhelpful to extremely helpful; and d) willingness to recommend the interventions to others on a 5-point scale ranging from strongly disagree to strongly agree. This information was collected from an administrative component of the website, nurse logs of contacts, and survey data."

6b) CONSORT: Any changes to trial outcomes after the trial commenced, with reasons

NA

7a) CONSORT: How sample size was determined

7a-i) Describe whether and how expected attrition was taken into account when calculating the sample size

The power analysis was described in the previous paper.
Duffy, S.A., et al., Protocol of a randomized controlled trial of the Tobacco Tactics website for operating engineers. BMC Public Health, 2012. 12(1): p. 335.

7b) CONSORT: When applicable, explanation of any interim analyses and stopping guidelines

NA

8a) CONSORT: Method used to generate the random allocation sequence

Not mentioned how to generate the random allocation sequence. We just stated that "One hundred forty five smokers were randomized to either the Tobacco Tactics website with nurse phone counseling and access to nicotine replacement therapy (NRT) or to the 1-800-QUIT-NOW telephone line which provided an equal number of phone calls and NRT"

8b) CONSORT: Type of randomisation; details of any restriction (such as blocking and block size)

"Since there was a high probability of cross-contamination within training sessions, randomization occurred at the training class level rather than individual level "

9) CONSORT: Mechanism used to implement the random allocation sequence (such as sequentially numbered containers), describing any steps taken to conceal the sequence until interventions were assigned

Not mentioned.

10) CONSORT: Who generated the random allocation sequence, who enrolled participants, and who assigned participants to interventions

Not mentioned.

11a) CONSORT: Blinding - If done, who was blinded after assignment to interventions (for example, participants, care providers, those assessing outcomes) and how

11a-i) Specify who was blinded, and who wasn't

Participants were blinded after assignment to interventions and "Since there was a high probability of cross-contamination within training sessions, randomization occurred at the training class level rather than individual level "

11a-ii) Discuss e.g., whether participants knew which intervention was the "intervention of interest" and which one was the "comparator"

Not mentioned in the manuscript. But In the informed consent, we stated that "The purpose of this study is to test two different interventions to help people quit smoking." and not stated either "intervention of interest" or "comparator".

11b) CONSORT: If relevant, description of the similarity of interventions

"According to the recommendations for the design of control group conditions in clinical trials, the control group condition should be designed to be equivalent as much as possible on time spent, follow-up times, and attention given to participants. In keeping with the recommendations, the 1-800-QUIT-NOW intervention was chosen as a control condition since it was as equivalent as possible to the Tobacco Tactics website intervention in terms of baseline counseling from the study nurse, numbers of follow up calls, and medications available."

12a) CONSORT: Statistical methods used to compare groups for primary and secondary outcomes

"To compare the two interventions on efficacy and usage, $\chi 2$ tests or Fisher's exact tests and t-tests were conducted using an intention-to-treat analysis in which non-responders were considered smokers. These analyses for quit rates were repeated controlling for differences between the groups using logistic regression. Since the randomization occurred at the training group level, to tests for cluster effects, test of heterogeneity for smoking status at 30-day and 6-month follow-up were performed using mixed models"

12a-i) Imputation techniques to deal with attrition / missing values

NA

12b) CONSORT: Methods for additional analyses, such as subgroup analyses and adjusted analyses

"Since the randomization occurred at the training group level, to tests for cluster effects, test of heterogeneity for smoking status at 30-day and 6-month follow-up were performed using mixed models. Since the sample size was small, if there was no significant heterogeneity, further analyses were conducted with chisquare tests or t-tests not adjusting the clustering by training group."

RESULTS

13a) CONSORT: For each group, the numbers of participants who were randomly assigned, received intended treatment, and were analysed for the primary outcome

"leading to 67 participants in the website group and 78 participants in the 1-800-QUIT-NOW group (N=145)"

13b) CONSORT: For each group, losses and exclusions after randomisation, together with reasons

Retention rates were mentioned. "Eighty three percent of the sample (n=120) completed the 30-day survey and 73% (n=105) completed the 6-month survey."
Reasons for exclusion and for losses were mentioned in the CONSORT chart (Fugure 1).

13b-i) Attrition diagram

CONSORT chart (Fugure 1).

14a) CONSORT: Dates defining the periods of recruitment and follow-up

"Over the course of three years (2010 to 2012), "

14a-i) Indicate if critical “secular events” fell into the study period

NA

14b) CONSORT: Why the trial ended or was stopped (early)

NA

15) CONSORT: A table showing baseline demographic and clinical characteristics for each group

Table 1 describes "Baseline Characteristics of the Tobacco Tactics Website and 1-800-QUIT-NOW Participants"

15-i) Report demographics associated with digital divide issues

We reported age, education, gender, race,...

16a) CONSORT: For each group, number of participants (denominator) included in each analysis and whether the analysis was by original assigned groups

16-i) Report multiple “denominators” and provide definitions

We reported the number of participants included for each analysis (Table 1 -- Table 3).

16-ii) Primary analysis should be intent-to-treat

Not in Result section but in Methods section, we mentioned "using an intention-to-treat analysis in which non-responders were considered smokers"

17a) CONSORT: For each primary and secondary outcome, results for each group, and the estimated effect size and its precision (such as 95% confidence interval)

Not mentioned. We reported the results of $\chi2$ tests or Fisher’s exact tests and t-tests.

17a-i) Presentation of process outcomes such as metrics of use and intensity of use

Table 3 reports the usage of the interventions (e.g., contacts with the intervention, visits to the website, use of NRTs....)

17b) CONSORT: For binary outcomes, presentation of both absolute and relative effect sizes is recommended

We reported the results of $\chi2$ tests or Fisher’s exact tests and t-tests.

18) CONSORT: Results of any other analyses performed, including subgroup analyses and adjusted analyses, distinguishing pre-specified from exploratory

Mentioned in Methods section. "Since the randomization occurred at the training group level, to tests for cluster effects, test of heterogeneity for smoking status at 30-day and 6-month follow-up were performed using mixed models. Since the sample size was small, if there was no significant heterogeneity, final analyses were conducted with chi-square tests or t-tests not adjusting the clustering by training group"

18-i) Subgroup analysis of comparing only users

We did not perform subgroup analysis of comparing only users.

19) CONSORT: All important harms or unintended effects in each group

We reported harm reduction (e.g., number of cigarettes smoked, nicotine dependence score...)

19-i) Include privacy breaches, technical problems

NA

19-ii) Include qualitative feedback from participants or observations from staff/researchers

"Overall helpfulness of the phone calls was rated significantly higher in the website group than the 1-800-QUIT-NOW group (p=.023). Participants in the website group reported more comfort with asking questions (p=.010), more satisfaction with the answers provided by the counselors (p=.003), and felt more supported (p<.000) than those in the 1-800-QUIT-NOW group. While 70.1% of the website group received any kind of NRTs, only 5.1% of the 1-800-QUIT-NOW group received NRT (p<.000); patches (40.3% vs. 2.6%, p<.000), gum (59.7% vs. 1.3%, p<.000), lozenges (9.0% vs. 0.0%, p=.009), and patches and gum or lozenges (35.8% vs. 0.0%, p<.000). There was no difference between the groups in terms of recommending the intervention to someone else (p=.171)."

DISCUSSION

20) CONSORT: Trial limitations, addressing sources of potential bias, imprecision, multiplicity of analyses

20-i) Typical limitations in ehealth trials

Due to a high probability of contamination within training groups, we randomized training groups instead of individuals and kept the participants blinded.

21) CONSORT: Generalisability (external validity, applicability) of the trial findings

21-i) Generalizability to other populations

"The sample was primarily white and male, but was representative of the sample of Operating Engineers in Michigan"

21-ii) Discuss if there were elements in the RCT that would be different in a routine application setting

we randomized training groups instead of individuals and tried to control for the clustering effects within training groups.

22) CONSORT: Interpretation consistent with results, balancing benefits and harms, and considering other relevant evidence

22-i) Restate study questions and summarize the answers suggested by the data, starting with primary outcomes and process outcomes (use)

"The Tobacco Tactics website intervention for Operating Engineers produced three times higher quit rates at 30-day follow-up than the 1-800-QUIT-NOW group"

22-ii) Highlight unanswered new questions, suggest future research

", future interventions may need to combine behavior change strategies targeting weight loss with those targeting smoking cessation"

Other information

23) CONSORT: Registration number and name of trial registry

"Trial Registration: ClinicalTrials.gov NCT01124110"

24) CONSORT: Where the full trial protocol can be accessed, if available

"The protocol of this study was described in previously published manuscript "

Duffy, S.A., et al., Protocol of a randomized controlled trial of the Tobacco Tactics website for operating engineers. BMC Public Health, 2012. 12(1): p. 335.

25) CONSORT: Sources of funding and other support (such as supply of drugs), role of funders

"Funding source for the trial: Blue Cross Blue Shield of Michigan Foundation Grant Number N011646-1465.RFP & the National Institutes of Health (NIH) Grant Number 5R21CA152247-02"

X26-i) Comment on ethics committee approval

"Institutional Review Board approval was received from the University of
Michigan"

x26-ii) Outline informed consent procedures

"Operating Engineers interested in the study were provided with an information sheet about the study and consent forms"

X26-iii) Safety and security procedures

Not mentioned. We mentioned the security procedures in another paper.

Duffy, S.A., et al., Protocol of a randomized controlled trial of the Tobacco Tactics website for operating engineers. BMC Public Health, 2012. 12(1): p. 335

X27-i) State the relation of the study team towards the system being evaluated

"Conflicts of Interest: None declared."
